# Influence of Family Environment on the Scientific Fitness Literacy of Preschool and School Children in China: A National Cross-Sectional Study

**DOI:** 10.3390/ijerph19148319

**Published:** 2022-07-07

**Authors:** Xiang Pan, Huan Wang, Dongming Wu, Xinhua Liu, Pengyu Deng, Yanfeng Zhang

**Affiliations:** 1 China Institute of Sport Science, Beijing 100061, China; panxiang9804@163.com (X.P.); wanghuan@ciss.cn (H.W.); wudongming@ciss.cn (D.W.); liuxinhua@ciss.cn (X.L.); 2 Graduate School of Health and Sports Science, Juntendo University, Inzai 270-1695, Japan; deng@juntendo.ac.jp

**Keywords:** screen time, exercise, motivation, health, attitude

## Abstract

Scientific fitness literacy (SFL) is a holistic concept based on physical literacy but has a smaller scope and is more specific to exercise and scientific fitness. We developed an instrument to assess SFL and explored the differences and similarities in the effects of the home environment on children’s SFL. Data from a nationwide stratified random sample of children were analyzed using multiple linear regression. SFL scores were significantly lower for preschoolers than for school-age children. The family environment has an impact throughout the preschool and school years, with school-age children’s SFL being less influenced by family members than preschool children; screen time has a negative impact on their SFL but reducing the number of electronic devices and increasing parental physical activity and modeling can alleviate its impact. The economic status of the family is crucial, with sports consumption expenditure and household sports equipment ownership being favourable factors for children’s SFL. Positive parental attitudes and sporting habits have a positive impact on their children’s SFL. The findings of this study can be used to improve children’s SFL in the home environment and to take effective measures to avoid the risk factors.

## 1. Introduction

Countries worldwide face problems related to low levels of physical activity and fitness among children and adolescents [1,2,3], which has worsened with the onslaught of COVID-19 [4]. An international survey involving 16 million people showed that only 20% of young people experience the pleasure of sport and benefit physically and mentally from regular physical activity [1]. Childhood is a crucial stage in the formation of values, patterns of behavior, and habits that have a profound impact on an individual’s whole life course. Therefore, helping children establish the right concepts and habits has become a key theoretical and practical research area in the fields of education, sociology, and psychology. Numerous studies [5,6,7,8,9] have shown that the family environment has a significant impact on children’s and adolescents’ sport-related psychology, behavior, and habits. It plays an important role because: (a) parents are the first people with whom children are socialized [5,9,10] and (b) the family environment continues to influence children and adolescents throughout their formative years. School-based curricula are often insufficient to improve children’s exercise levels. Extracurricular physical activities are usually voluntary, and such activities make a crucial contribution to the total length and volume of exercise; however, when family and community interventions are added to school-based curricular interventions, children’s exercise levels far exceed those of school-based interventions alone [11]. Considering the widely established positive relationship between health and schooling achievements, an increased level of sports exercise can also lead to better educational outcomes of students [12,13,14].

To help uncover effective interventions for Chinese children’s sporting behaviors, habits, and cognitions, we have combined China’s National Fitness Program with cutting-edge international research relating to physical literacy to propose the concept of scientific fitness literacy (SFL) [15,16]. SFL refers to people’s ability to realize their embodied potential according to their respective talents; to actively interact with their environment; to build a virtuous cycle from motivation, knowledge and understanding, attitude, ability, and skills to behavior and habit; and ultimately to be able to consciously engage in sport-related activities throughout their lives. The concept of “embodiment” [15,16,17] in SFL calls for a transformation of traditional perceptions of exercise and physical activity from the previous emphasis on instrumentalized views (sport as an end) to understanding the essence of fitness (essential joy of sport), from focusing solely on the outcomes of exercise (e.g., physical indicators) to how exercise shapes the whole organism (motivation, attitudes, habits, skills, and environmental motivation), from one-size-fits-all evaluation criteria (external standards) to a diverse evaluation system. Monotonous interventions need to become differentiated and segmented. Compared to physical literacy, SFL is based on physical fitness and covers a smaller range of activities, including only scientific fitness based on the dimensions of health and exercise, rather than the full range of lifestyles based on a broader basis of physical activity.

In the context of SFLs integration of the social–ecological model and self-determination theory, various factors—external, environmental, and human—are seen to influence human behavior directly or indirectly. In our context, the family environment is the main external factor influencing the SFL of children from the perspective of social–ecological theory [18,19,20]. The family environment covers a wide range of subcomponents, each of which plays a different role at different stages of a child’s development. According to a literature review conducted by Li et al. [21], the family environment is divided into modeling, involvement, encouragement and persuasion, instrumental support, and restriction. In addition to these behavioral factors, parents’ education level, salary, primary caregiver status, and other family characteristics are also key factors. People go through a process of transition towards a larger life setting during childhood, from preschool to primary education [22,23]. Compared to the unorganized quality of preschool education, characterized by high parental involvement and less curricular input [24], children entering elementary school for systematic schooling undergo a shift in their life scenario, education methods, and the role of the primary instructor [23,25]. This will inevitably have various effects on the influence of the home environment on SFL. Few studies have examined the effects of changes in children’s life environments on physical literacy during this period, and considering that SFL has a more precise assessment of exercise and fitness, studies focusing on the effects of family environment on SFL in preschool and school-aged children are almost empty.

The objective of this study is therefore to develop a reliable and valid indicator system for the assessment of children’s SFL to quantify this literacy; subsequently, we develop a multiple regression model of the effect of home environment factors on the SFL scores of preschool and school children to investigate the similarities and differences between the effects of the home environment on the SFL of preschool (3–6 years old) and school children (7–9 years old). These theoretical underpinnings can provide parents with a number of intervention perspectives to improve their children’s SFL from the home environment, contributing to the enhancement of children’s sports participation at this stage and into the future and promoting their scientific fitness ability from attitudes to habits.

## 2. Materials and Methods

### 2.1. Sampling and Grouping

The analysis covered children aged 3 years old in 471 counties (districts) in 31 provinces in China. A total of 2 counties (districts) in each province were randomly selected, with one child aged 3–6 years old and one child aged 7–9 years old in each county. Finally, the sample comprised an estimated 1884 children. The survey covers all provinces of mainland China and is very widely distributed. Chinese children are well represented across the country. 

To explore differences in how home environment influences SFL before and during school, children were divided into a preschool group (3–6 years) and a school group (7–9 years) for the following reasons. Children’s physical and mental development and dependence on their family changes in stages: for preschool children (3–6 years old), home is their main socialization contact environment. Children aged 7–9 years begin systematic schooling and have frequent contact with people other than their parents. Though they remain dependent, they gradually gain some degree of independence from their parents.

As preschool children are illiterate and unable to express themselves fully, the questionnaire was mainly answered by their parents based on daily observations. As preschool children have acquired some simple vocabulary, they can answer simple questions while their parents can answer complex questions; parents can help children answer abstract questions. Therefore, we divided the questionnaire into three parts: answered by children, parents, and by children with parental help.

### 2.2. Evaluation of SFL Indicators

**Figure 1:**
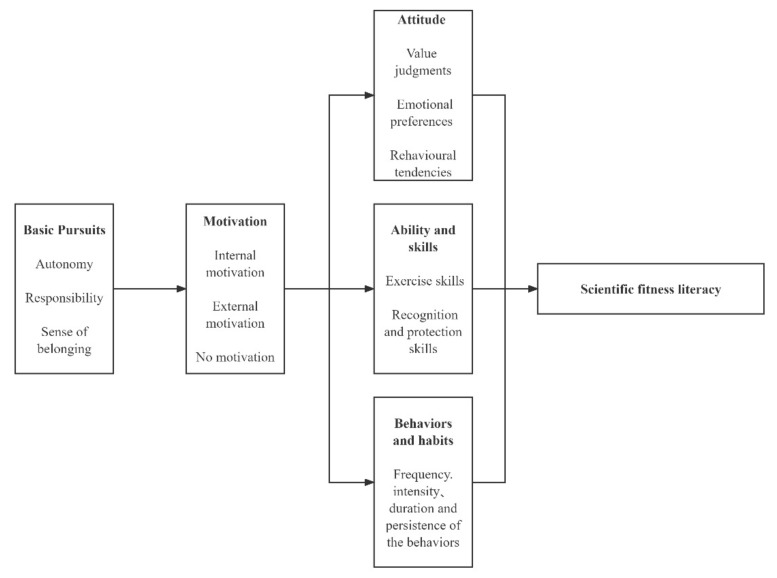
Measurement model of scientific fitness literacy.

Behavioristic psychology believes that motivation is the starting point of human behavior. According to the self-determination theory [17,26,27]—a prominent current theory in motivation research—motivation is required to meet the basic needs of human beings (autonomy, sense of competence, and belonging). Individuals grow in the process of meeting their basic needs. Motivation has effects on multiple dimensions, including cognitive, emotional, physical, and behavioral dimensions [28,29]. In this context, we adopted attitude (emotional dimension), ability and skills (physical dimension), and behavior and habits (behavioral dimension) as the three dimensions (latent variables) reflecting SFL. Figure 1 shows the measurement model we constructed for SFL. Attitude includes three sub-items, namely value judgments, emotional preferences, and behavioral tendencies; the ability and skills dimension includes two sub-items, namely exercise skills and recognition and protection skills; and the behavior and habits dimension includes one sub-item, which includes measurements of the frequency, intensity, duration, and persistence of exercise behavior. Each sub-item corresponds to one scale. Detailed dimensions, sub-items, and examples of questions are shown in Table 1.

### 2.3. Family Environment Indicators

Based on the findings of Sallis et al. [30], we divided family environment into two aspects, namely, basic information on the family environment and family behavior. Basic information on the family environment includes three indicators: parents’ education, parents’ income level, and whether the parents are the primary caregivers. Family behavior includes five indicators, which are modeling, involvement, encouragement and persuasion, instrumental support, and restriction.

### 2.4. Procedures

Full-time researchers from selected municipal sports administrations were trained to familiarize them with the specific program of this study. The information of the interviewees at the monitoring points came from the local statistical bureau. Respondent information was obtained from local statistical offices, and a random sample was selected to meet the requirements. After contacting them by telephone and obtaining their permission, the information was filled in the questionnaire after face-to-face interviews in the community of the designated study site, lasting approximately one hour per participant in each interview, while quality control was performed by return telephone calls and field notes during the survey. The duration of the survey was from June to August 2020.

Names were replaced by Arabic numerals in this study, the entire recall sample contained no identifiable personal information, and the private information of all individuals was protected. Before the study began, participants provided written consent to participate after receiving information about the procedures and purpose of the study. Full ethical approval was obtained from the China Institute of Sport Science, Beijing, China (CISS-2019-10-29).

### 2.5. Analysis Methods

In this study, a nationwide stratified random sample of children from 31 provinces (autonomous regions and municipalities) and the Xinjiang Production and Construction Corps was conducted. SPSS19.0 (IBM Corp., Armonk, NY, USA) and AMOS22.0 (IBM Corp., Armonk, NY, USA) were used for data analysis.

**Figure 2:**
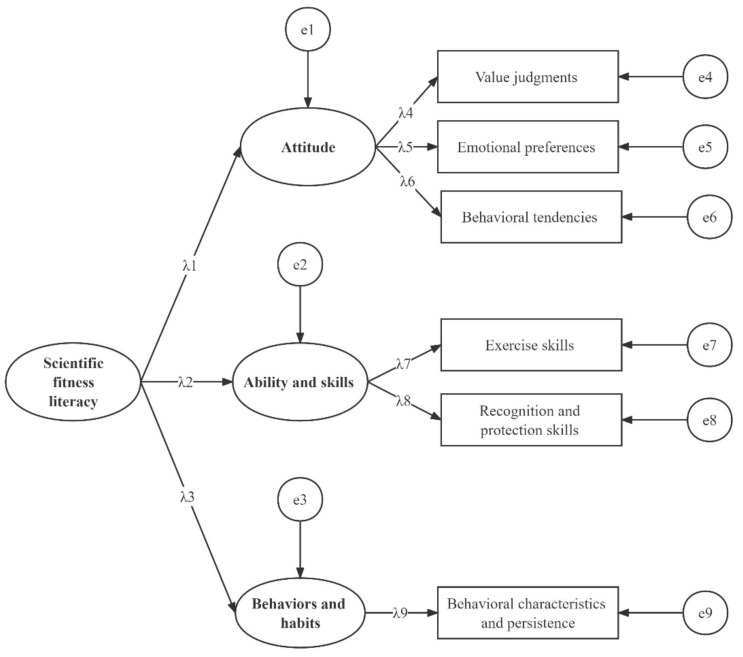
Confirmatory factor analysis model of scientific fitness literacy.

In this study, Cronbach’s alpha coefficients were used to assess the reliability of the indicators. The overall goodness of fit, composite reliability, and convergent validity of the indicators were analyzed using confirmatory factor analysis (CFA) based on structural equation modeling. Root mean square error of approximation (RMSEA) and adjusted goodness-of-fit index (AGFI) are used to test the overall goodness of fit of the model. The measurement model is shown in Figure 2. 

Based on the questionnaire responses, we constructed the SFL and family environment indicators. After examining the reliability and validity of SFL in different age groups, multiple regression models were constructed for each age group to analyze the influence of family environment on SFL. The best fitting models for different age groups and different family environment sub-influencing factors were assessed using stepwise multiple regression analyses.

## 3. Results

### 3.1. Descriptive Statistics

The estimated sample size for both age groups was 1884 (942 for each group), and the actual sample size was 1716, resulting in a recovery rate of 91.1%. The number of valid responses recovered for each age group was 832 for preschool children (3–6 years old, M = 391, F = 441) and 884 for school children (7–9 years old, M = 407, F = 477). The urban–rural distribution of each age group in this survey was almost equal. Meanwhile, the urban–rural distribution was slightly uneven in the preschool group, but there were no significant gender or regional differences in the SFL and its subscales for either age group (*p* > 0.05). The SFL indicators and several of its sub-dimension scores (all percentages) demonstrated significantly higher scores for school children than for preschool children (Table 2).

### 3.2. Reliability Test

The reliability of the three major dimensions of SFL in the two sets of questionnaires were all within the acceptable ranges. The Cronbach’s alpha coefficients for ability and skills and behavior and habits in the 3–6 years group were 0.740 and 0.543, respectively; for the three dimensions of attitude, ability and skills, and behavior and habits in the 7–9 years group, the coefficients were 0.614, 0.781, and 0.532, respectively. The reliability of the behavior and habit dimension is slightly lower for school children. The Cronbach’s alpha coefficients for the overall questionnaire for the 3–6 and 7–9 year old groups were 0.543 and 0.532, respectively. As this questionnaire involves various indicators including different scenarios (in school and out of school) and the frequency, intensity, duration, and persistence of exercise, the correlations are relatively weaker. Table 3 reports the correlation coefficients between the scores of each dimension of SFL for children in both age groups. We did not design a questionnaire corresponding to the attitude dimension for preschool children because we considered this personal subjective feeling to be too advanced for preschool children to understand or express.

### 3.3. Confirmatory Factor Analysis: Overall Goodness of Fit, Composite Reliability, and Convergent Validity of the Model

The overall goodness of fit of the measurement models for SFL was good for each age group (3–6 years group: RMSEA = 0.066 (≤0.08), AGFI = 0.953 (≥0.9); 7–9 years group: RMSEA = 0.056 (≤0.08), AGFI = 0.947 (≥0.9)). The reliability of the two models was high, with composite reliability in the ranges of 0.63–0.79 and 0.66–0.80 for the preschool and school children, respectively. The convergent validity of the measures is acceptable (average variance extracted ≥0.5), except for the attitude dimension for preschool children. Nonetheless, we retained the attitude dimension because it is an important SFL dimension with extensive theoretical and practical significance, and its average variance extracted was not far from the acceptance threshold.

### 3.4. Multiple Stepwise Regression Analysis

After testing for reliability and validity, we used SFL as the dependent variable, and all the previously studied family environment sub-factors (Table 4) as independent variables to build multivariate linear regression models for each group. We used stepwise regression analysis to screen all family environment sub-factors to obtain the best fit model for each group and analyze which family environment sub-factors play a major role. The screening criterion for the stepwise regression was that the contribution of each family environment sub-factor to SFL was entered into the model in the descending order; the entry criterion of sub-factors was *p* < 0.05 and the deletion criterion was *p* > 0.1.

### 3.5. Regression Model for Preschool Children

The regression model for preschool children demonstrated a high degree of fit (Table 5). The sub-factors of family environment have a high explanatory power for SFL (adjusted R^2^ > 0.5).

Seven factors were included in the model for preschool children. At the 3–6-year-old stage, sports facilities and equipment owned by the family had the largest effect on SFL (β = 0.264, *p* < 0.001). The length of time parents spent with their children on holidays also influenced the SFL of preschool children. Furthermore, higher maternal and grandparental physical activity participation, annual household income, and caregiver ratings of child activity and activity capacity had a positive effect on SFL in preschool children. 

### 3.6. Regression Model for School Children

Among school children (Table 6), the influence of the number of home sports facilities and equipment on the child’s SFL was strongest (β = 0.399), followed by parental encouragement (β = 0.163). Many factors were included in the model, with the father’s education and parents’ sport-related financial investment, exercise accompaniment, sporting habits, and evaluation of the child’s activity level having an impact on the child’s SFL (β range: 0.084–1.117, *p* < 0.001). Children’s SFL was significantly negatively influenced by the amount of time spent watching television on school days, with the second highest absolute standardized coefficient size.

### 3.7. Regression Model of Screen Time for School Children

Considering that school children’s SFL is negatively affected by screen time, we used screen time as the dependent variable and other home environment subfactors as independent variables to build a multiple linear regression model as shown in the Table 7 (F-test = 39.080, *p* < 0.001, adjusted R^2^ = 0.232, exhibiting fair explanatory power). A total of seven sub-factors entered the model, with the highest absolute value (β = 0.265) and positive direction of the standardized coefficient for the number of electronics owned by the household. The direction of parental screen time use is also positive. There were five sub-factors with a negative direction: father (β = −0.107) and mother (β = −0.152) on frequency of accompanying children outdoors and exercising themselves (β = −0.075), willingness to pay for children’s sports consumption (β = −0.113), and distance to outdoor facilities, parks, etc., around the home (β = −0.139); in other words, these factors negatively affected children’s screen time use.

## 4. Discussion

The sample collected for this study covered all provinces of the country and was analyzed for differences in the sub-factors of the family environment affecting SFL in preschool and school children. SFL values were significantly higher for school children than for preschool children. The home environment subfactors and their importance varied with age. For school children, SFL was negatively but remedially affected by screen time, and parental involvement and example remained an important part, not only in terms of accompanying and encouraging sports participation, but also in terms of improved screen time. The influence of fathers is gradually increasing. The most important point is that the economic base of the family greatly influences the SFL of preschool and school-age children, both in terms of the correlation between a high SFL and high sports equipment ownership; at the same time, investment in sports consumption, such as sports training, is also crucial. These findings enhance our understanding of the ways in which home environmental factors contribute to SFL in preschool and school-age children, so that we can better target our efforts to improve children’s SFL in the home environment and take effective measures to avoid risk factors. These parental interventions to increase children’s SFL on the family environment can contribute to enhancing children’s sports participation at the current stage and into the future and promote their scientific fitness ability from attitudes to habits.

### 4.1. Variability in Children’s SFL

There were significant differences in SFL between school-age and preschool children. School children scored high on the attitude dimension, had more developed cognition and attitudes, and were able to express their positions and value judgements more independently; moreover, the influence of personal socialization became apparent. We found that the total SFL score, as well as scores on the other two dimensions, were significantly higher for school-aged children than for preschool-aged children, with the exception of the effect of the unmeasurability of the attitude dimension for preschool children. In a large study (with a good representative sample size of 10,034) of Canadian children aged 8–12 years, an increasing trend in physical ability scores and knowledge and understanding scores with increasing age was found, with no age differences in motivation and confidence scores [31]. While the aforementioned study differs slightly from our study, there is an overlap in these dimensions, which to some extent corroborates our results. No gender or urban–rural differences were shown within either group for either preschool or school-age children. However, this may be incongruent with other studies, which suggest that non-metropolitan area youth aged 12–17 years are more likely to be involved in sports, particularly males, and that overall sports participation is higher among females than males [32].

### 4.2. Importance of Material Support and Economic Conditions of the Family for Children’s SFL

The analysis revealed that the largest absolute values of the standardized multiple regression coefficients for “Number of sports facilities and equipment owned by the household” in the models for children aged 3–6 and 7–9 years, material support was dominant in all family environment sub-factors regardless of whether parents were primary caregivers for their children. This did not change with the transition to school life: the more sports equipment a household has, the higher the individual’s SFL. Material support involves undermining the spatial and instrumental limits on children’s physical activity, by providing them with the maximum support for physical activity; the greater the family’s ability to provide support, the better. Sports/exercise participation was higher for youth who had at least 3–4 items of sports/exercise equipment at home [33]. Furthermore, the amount of physical activity equipment at home was positively and negatively correlated with physical activity and television viewing time, respectively, for children [20,34]. We therefore believe that acquiring more sports equipment in the home is likely to be an effective way of increasing SFL in both preschool and school-age children; however, this may involve the financial level of the family. Annual household income is included in the model for preschool children; we found that the family’s finances can have a positive impact on a preschooler’s SFL. Gorely et al. found that not only girls from low socioeconomic families, but also those living in low socioeconomic neighbourhoods may be at an increased risk of reduced participation in sports and exercise [35]. As for school-age children, economic conditions seem to have changed its expression. In the model for school-age children, “parents’ willingness to pay for their child’s sports-related training” replaces “family income” in the model. Overall, we found that the higher the parental support level, the higher the child’s physical activity participation. There is even a link to obesity in preschoolers based on economic conditions, with children from poorer families having a higher incidence of low sports participation and obesity [36,37]. The importance of the family’s financial base is crucial for children’s SFL, both in terms of the purchase of sports equipment and support for the child’s sports training. A study on 11–15 year olds found that children from wealthy families are more likely to participate in organized sports, and the gap tends to increase with age [38]. The group targeted in our study was children aged 3 to 9 years, and the results revealed an effect of household economic level on children’s SFL, which may expand with age. This study provides parents with many perspectives on interventions for children’s SFL in the home environment, such as increasing parental sports participation, the number of sports equipment owned by the family, and sports consumption.

### 4.3. Negative Impact of Screen Time on SFL in School Children and Preventive Measures

This study also shows that screen time is starting to consume children’s out-of-school time: new activities begin to compete for their leisure time. Children’s screen time (television and various electronic devices) is among the most influential sub-factors, and parental sports participation has a diametrically opposed effect on children’s SFL to television viewing time. With China’s rapid economic development, the increasing popularity of smart devices has increased children’s screen time. Studies have shown that excessive screen time increases children’s sedentary time, limits their physical activity levels, and reduces their probability of participating in sports-related clubs or interest groups [20,39]; it also increases the risk of obesity [40]. The presence of a television in the bedroom and corresponding parental behavior patterns were significantly and positively associated with children watching television [41]. Hence, it has been suggested that children should not have televisions in their bedrooms and that families should set limits on television viewing by, for example, banning television during meals [42]. 

We developed a multivariate regression model of screen time to explore measures of school children to reduce screen time in the home environment. We found that two factors contributed to school-age children’s screen time, with the largest coefficient of quantification of the number of electronic items in the home, followed by parental screen time. A parental screen time greater than 2 h per day was the determining factor for children’s screen time [43]. A study in Fiji found that approximately 24% believed there was no negative impact on children due to screen time and more than half (56%) of the parents (or caregiver guardians) thought that screen time for children below the age of 2 years actually has positive consequences on children. Of these, the majority (76.7%) think that screen time makes their children smart or helps them to learn from a very young age [44]. We found that parental accompaniment of sport, attitudes towards sports consumption, and the frequency of their own physical activity had an inverse effect on the length of time children spent on screen. A study by He et al. suggests that children’s extended screen time stems from a lack of control over the home environment by schools and a perception that parents were poor role models and stresses the need for parents to play a key role in reducing their children’s screen-related sedentary behaviour and increasing their physical activity levels. Notably, the location of the home also appears in the models of our results, and that proximity to places where exercise is available may reduce children’s screen time, with headmasters of 14 elementary schools in London and Middlesex County, Ontario, indicating that limited gym resources/space within the school increased students’ screen time [45]. Dadvand et al. suggest that an increase in greenness in residential surroundings was associated with 11–19% lower relative prevalence of overweight/obesity and excessive screen time [46]. According to our comprehensive research, parents can take measures such as reducing the number of electronics in the home, increasing sports companionship, or choosing an appropriate home address, etc., to reduce the negative effects of children’s screen time on school-age children’s SFL.

### 4.4. Complexity and Variability in Parental Influence on Children’s SFL

Comparing the two multiple regression models for children aged 3–6 years and 7–9 years, we found an interesting pattern in which grandparental exercise participation and parental holiday companionship had some influence before school admission, but parental weekend exercise participation took over once children started at elementary school, and time spent watching television on school days began to influence children’s SFL. This may be because parents and grandparents take care of children together during the preschool period and spend more time with them, while parents’ participation in sports and accompaniment during holidays becomes more important during schooling. Nevertheless, this can be seen as a changing pattern of intergenerational influences. It has also been shown that there are gender differences in parental influence: girls’ sports participation can be predicted by the exercise habits of their fathers and mothers, while sons’ physical activity can only be predicted by the regular physical activity of their fathers [47]. 

Both parental involvement and companionship have a positive effect on children’s participation and interest in sports: children have a greater chance of high physical activity participation when parents participate in sports at least 1–2 times per week. This is supported by the study of Hungarian preschoolers, by Müller et al. [37], who found that obese children were significantly less likely to participate in physical activity with their parents once a week than normal weight children. During the preschool stage, mothers generally act as role models and companions. Therefore, the mother’s care and attention gives the child security to face unfamiliar situations. Once enrolled in school, the father’s educational philosophy and joint engagement play an important role. Numerous psychological studies [48] have shown that the father’s role begins to increase in importance between the ages of 6 and 7 years, as fathers help children develop an independent personality as well as positive qualities such as confidence and courage. Table 4 showed that this paternal role applies to SFL. As children are in a critical period of forming ideas and values, their fathers’ education level reflects differences in their own ideas and attitude towards physical exercise, leading to children forming different value judgements and emotional attitudes towards physical activity and exercise. Hence, their involvement as guides and motivators for children who are still at the stage of overcoming difficulties and acquiring skills is more pronounced [21]. A study by Huppertz et al. on Dutch and Finnish twins found that the mean in exercise behavior tended to be higher in children of highly educated parents [49]. Although it has been suggested that there is an association between parental exercise and participation in older (15–16 years) but not younger adolescents (13–14 years), for the purpose of this study’s results, parents influence their children’s sports participation, both as accompanying sports participants and simply as supporters of sports habits [47].

### 4.5. Strengths and Limitations

This study is based on a national survey that provides a comprehensive coverage of mainland China, using a stratified random sample, questionnaires, and quantitative analysis of SFL and most household environmental factors. Although the SFL study is smaller in scope than the physical literacy study, it is also a more in-depth assessment of fitness attitudes and perceptions, fitness skills, and fitness habits, enriching the theoretical basis of physical literacy.

As the study population involved preschool children and the survey method was a questionnaire, it was difficult to achieve accurate measurements in terms of subjective attitudes towards fitness, resulting in an insufficient reliability in measuring the attitude dimension when measuring SFL. In this study, to reduce its influence in the analysis of the results, attitude was not included in the scores for preschool children. Thus, we were only able to construct a model of the influence of family environment on SFL and its sub-dimensions for the two groups of children separately and subsequently compare them. Although we have set both the SFL and the scores for each dimension as percentages to moderate these effects, it is still not possible to completely avoid the reduction in the scientific validity of the comparison between preschool and school children. Another limitation is that although this study is a comprehensive survey covering mainland China, there are many regions in mainland China, and the sample size of individual regions is not sufficient to ensure the accuracy of the study results. Therefore, we will consider expanding the sample size of individual provinces to enrich the theoretical basis of SFL by selecting representative provinces for subsequent studies.

## 5. Conclusions

We have found that family environment is influential throughout preschool and school stages. School-age children’s SFL are less influenced by family members than preschoolers and more by other family environmental factors. Screen time has a negative impact on their SFL but reducing the number of electronic devices, increasing parental physical activity and modeling, and having physical activity areas around the home can reduce their impact. The economic condition of the household is particularly important, especially among school-age children, with sports consumption expenditure and household sports equipment ownership being the favourable factors for children’s SFL, with the latter playing a more significant role. Parents have a strong influence on their children’s SFL, as their positive attitude and good exercise habits not only have a positive impact on their children’s SFL but can also indirectly be a protective factor for their children’s SFL by reducing the negative effects of screen time.

## Figures and Tables

**Table 1:** Dimensions, sub-items, and questionnaire examples for measuring scientific fitness literacy.

Group	Dimension	Sub-Item	Examples of Questionnaire Content
Preschool children	Ability and skills	Exercise skills	What do you think are the sports activities (including playing) that your child does regularly?
			What physical activities do you think your child is good at?
		Recognition and protection skills	How do you think your child’s current activity level compares to that of his or her peers?
	Behavior and habits	Frequency, intensity, duration, and persistence of exercise behavior	What is the average amount of time your child spends playing sports?
			What is the amount of time your child spends outside for physical activity on rest days?
School children	Attitude	Value judgments	What do you think are the advantages or disadvantages of participating in sports activities?
		Emotional preferences,	Do you like to play at school or go out with your family?
			Are you satisfied with your performance in physical education classes at school?
		Behavioral tendencies	Do you often go outside to participate in various activities (including playing) and exercise?
	Ability and skills	Exercise skills	What are your favorite physical exercise programs?
			What physical exercise programs have you tried?
		Recognition and protection skills	Do you continue to do the skills and knowledge you learned in school physical education outside of class or outside of school?
			Do you want to do a proper preparation activity before exercise?
	Behavior and habits	Frequency, intensity, duration, and persistence of exercise behavior	In most cases, other than physical education classes, what is the approximate duration of each physical activity (e.g., extracurricular physical activity, off-campus physical activity) that you perform?
			On average, how many days a week do you participate in physical activity (including extracurricular physical activity and out-of-school physical activity) in the last 1 month, excluding school physical education classes?

**Table 2:** Descriptive statistics and means of scores on scientific fitness literacy (SFL) and its sub-dimensions.

Group			N (Percentage)	SFL	Attitude	Ability and Skills	Behavior and Habits
Preschool children	Sex	Male	391 (47.0)	33.5 ± 12.8		19.2 ± 15.0	48.0 ± 17.1
		Female	441 (53.0)	34.1 ± 12.9		19.3 ± 13.9	49.1 ± 17.5
		*p*		0.560		0.986	0.351
	Area	Urban	564 (67.8)	33.3 ± 13.0		18.8 ± 14.5	47.9 ± 17.4
		Rural	268 (32.2)	35.0 ± 12.4		20.2 ± 14.2	50.0 ± 17.1
		*p*		0.067		0.199	0.105
	Total		832	33.8 ± 12.8		19.2 ± 14.4	48.6 ± 17.3
School children	Sex	Male	407 (46.0)	50.1 ± 13.6	69.2 ± 16.2	28.5 ± 22.5	52.6 ± 14.9
		Female	477 (54.0)	49.2 ± 14.0	67.8 ± 16.8	29.1 ± 22.6	50.8 ± 13.8
		*p*		0.334	0.200	0.685	0.058
	Area	Urban	525 (59.4)	49.6 ± 12.5	68.9 ± 14.9	28.1 ± 21.5	51.9 ± 13.7
		Rural	359 (40.6)	49.8 ± 15.5	68.0 ± 18.6	30.0 ± 23.9	51.4 ± 15.3
		*p*		0.883	0.430	0.213	0.578
	Total		884	49.7 ± 13.8 **	68.5 ± 16.5	28.8± 22.5 **	51.7 ± 14.4 *

* *p* < 0.05; ** *p* < 0.01. SFL = scientific fitness literacy.

**Table 3:** Correlation of total scientific fitness literacy score and its sub-dimensions.

Age Group	Items	SFL	Attitude	Ability and Skills	Behavior and Habits
Preschool children	SFL	1		0.747 **	0.833 **
	Ability and skills	0.747 **		1	0.257 **
	Behavior and habits	0.833 **		0.257 **	1
School children	SFL	1	0.710 **	0.849 **	0.733 **
	Attitude	0.710 **	1	0.358 **	0.332 **
	Ability and skills	0.849 **	0.358 **	1	0.467 **
	Behavior and habits	0.733 **	0.332 **	0.467 **	1

** *p* < 0.01; SFL = scientific fitness literacy.

**Table 4:** Sub-factors of influence of family environment and definitions.

Sub-Factors		Definitions
Basic home information	Father’s education	Select the father’s highest qualification.
	Mother’s education	Select the mother’s highest qualification.
	Parents’ income level	Select the option closest to the household income level.
	Whether the parents are primary caregivers	Whether the parents are child’s primary caregivers.
Family behavior	Modeling	Frequency of parental physical activity/What do parents often do on holidays?
	Involvement	Whether your parents participate in sports with you.
	Encouragement and Persuasion	Do your parents encourage and support your participation in physical activity?/Are your parents willing to listen to your ideas and opinions?
	Instrumental Support	What are the items you regularly use at home? (including sporting goods, electronics, etc.)
	Restriction	What requirements, restrictions, or rules do your parents have for your physical activity?

**Table 5:** Table of multiple regression models for the effect of home environment subfactors on preschool children’s scientific fitness literacy.

F-Test	Adjusted R^2^	Sub-Factors Entering the Model after Screening	Direction	*T*-Test	β	*p*
23.057 (*p* < 0.001)	0.271	Number of sports facilities and equipment owned by the household.	+	6.086	0.264	<0.001
		Parents’ comments on the amount of activity for their child.	+	4.063	0.184	<0.001
		Mothers often engage in physical activity.	+	2.535	0.119	0.005
		Time with parents during holidays.	+	3.476	0.149	<0.001
		Annual household income level.	+	2.604	0.110	<0.001
		The elderly in the family often engage in physical activity.	+	2.362	0.111	<0.001
		Parents’ assessment of their child’s capacity for physical activity.	+	2.028	0.092	0.009

Direction is the relationship between home environment sub-factors and SFL, with + indicating a positive correlation.

**Table 6:** Table of multiple regression models for the effect of home environment subfactors on school children’s scientific fitness literacy.

F-Test	Adjusted R^2^	Sub-Factors Entering the Model after Screening	Direction	*T*-Test	β	*p*
59.064 (*p* = 0.001)	0.516	Number of sports facilities and equipment owned by the household.	+	10.942	0.399	<0.001
		Parents always encourage their children to participate in physical activities and exercise.	+	4.150	0.163	<0.001
		Parents’ willingness to pay for their children to take sports-related classes.	+	2.894	0.108	<0.001
		Screen time on school days.	−	−4.463	−0.164	<0.001
		Whether the father is willing to accompany the child in sports activities.	+	3.140	0.117	<0.001
		Parents’ lifestyles during holidays (do they do a lot of sports-related activities?).	+	3.143	0.111	<0.001
		Parents’ assessment of their child’s capacity for physical activity.	+	2.588	0.091	<0.001
		Father’s education.	+	0.091	0.084	0.002

Direction is the relationship between home environment sub-factors and SFL, with + indicating a positive correlation and − indicating a negative correlation.

**Table 7:** Regression model of screen time for school children.

F-Test	Adjusted R^2^	Sub-Factors Entering the Model after Screening	Direction	*T*-Test	β	*p*
39.080 (*p* < 0.001)	0.232	Number of electronic items owned by households.	+	8.885	0.265	<0.001
		Does the mother accompany the child for outdoor activities?	−	−4.422	−0.152	<0.001
		Score for distance from home to park.	−	−4.547	−0.139	<0.001
		Parents’ willingness to spend money on their children’s sports and exercise.	−	−3.587	−0.113	<0.001
		Does the father accompany the child for outdoor activities?	−	−3.110	−0.107	0.002
		Parental screen time.	+	3.220	0.096	0.001
		Frequency of physical activity by parents.	−	−2.416	−0.075	0.016

Direction is the relationship between home environment sub-factors and screen time, with + indicating a positive correlation and − indicating a negative correlation.

## Data Availability

The data presented in this study are available on request from the corresponding author. The data are not publicly available due to privacy.
